# Supplementary material for: Transcriptomic response of skeletal muscle to acute aerobic versus combined exercise in chronic kidney disease
Source: PLoS One. 2026 Feb 25;21(2):e0324303. doi: 10.1371/journal.pone.0324303 (PMC12935244; doi:10.1371/journal.pone.0324303)
Supplement: S1 Table — Table represents the log fold change values from baseline to 24h following an unaccustomed session of aerobic exercise (AE), together with a brief description of known function. Abbreviations: fc, fold change. (DOCX) [file pone.0324303.s001.docx]

Table S1. Top 20 downregulated gene following AE

| Gene Symbol | Gene name | Gene Function | Log2fc | Adj P value |
| --- | --- | --- | --- | --- |
| GRIN2A | Glutamate ionotropic receptor NMDA type subunit 2A | A **subunit of the NMDA receptor**, which plays a central role in **neurotransmission** and **synaptic plasticity** in the **central nervous system. In muscle, it** could indirectly influence **muscle activation** through its role in controlling **motor neuron excitability**. | -5.9 | 0.001 |
| GPR1 | G protein-coupled receptor 1 | A receptor for **chemerin**, an adipokine involved in inflammation, metabolism, and immune responses. In muscle it may be in volved in the control of metabolism and mediating inflammatory pathways. | -5.9 | 0.0001 |
| TIGD4 | tigger transposable element derived 4 | A gene encoding a protein related to transposable elements, which are DNA sequences capable of moving or copying themselves to new positions within the genome. It does not have a well-documented role in muscle. | -5.5 | 0.02 |
| VSNL1 | visinin like 1 | A **calcium-binding protein** that belongs to the **neuronal calcium sensor (NCS) protein family. In muscle it may have roles in calcium signalling and excitation-contraction coupling.** | -4.9 | 2.40E-05 |
| OGDHL | oxoglutarate dehydrogenase like | A mitochondrial enzyme that is closely related to **oxoglutarate dehydrogenase**, a key enzyme in the **tricarboxylic acid cycle. It has key roles in energy production in muscle.** | -4.9 | 0.01 |
| PAX5 | paired box 5 | A transcription factor primarily known for its role in **B-cell development** and the regulation of genes necessary for the differentiation and maintenance of B lymphocytes. Its role in muscle is unclear. | -4.6 | 0.007 |
| FAM181A | family with sequence similarity 181 member A | Precise biological functions are not yet fully elucidated | -4.6 | 0.03 |
| C1orf158 | chromosome 1 open reading frame 158 | **Also known as NOA1 (Nitric Oxide-Associated 1)**, encodes a protein localized in mitochondria. It is involved in several fundamental cellular processes, primarily **mitochondrial function** and **ribosomal biogenesis. In muscle it s**upports **mitochondrial ribosome assembly and e**nsures proper mitochondrial translation and protein synthesis, crucial for oxidative phosphorylation. | -4.5 | 0.01 |
| DACT2 | dishevelled binding antagonist of beta catenin 2 | Involved in modulating **Wnt signaling. In muscle it plays a role in myogenesis.** | -4.4 | 0.01 |
| GPR61 | G protein-coupled receptor 61 | An **orphan G protein-coupled receptor with no known role in muscle** | -4.4 | 0.001 |
| ABCC6 | ATP binding cassette subfamily C member 6 | A transporter protein highly expressed in liver. Its role in muscle is unknown. | -4.4 | 6.8E-08 |
| HPSE2 | heparanase 2 | Has a role in regulating ECM components and has been linked to processes such as **inflammation**, **tissue remodeling**, and **development**. | -4.3 | 0.02 |
| MALRD1 | MAM and LDL receptor class A domain containing 1 | Involved in several cellular processes, including inflammation, cell signaling, and immune responses. In muscle it may play a role in the inflammatory response to injury. | -4.3 | 0.01 |
| TSHR | thyroid stimulating hormone receptor | A cell surface receptor that binds **thyroid-stimulating hormone**, produced by the **pituitary gland** that regulates the function of the thyroid gland. In muscle, thyroid hormones are essential for regulating **muscle energy production** and metabolism. | -4.3 | 0.01 |
| LMX1A | LIM homeobox transcription factor 1 alpha | A **transcription factor** that plays a key role in regulating the expression of genes involved in development, differentiation, and tissue patterning. In muscle it is thought to act as a **regulator of myogenesis.** | -4.3 | 0.01 |
| CHAD | chondroadherin | Involved in cell signaling, transcriptional regulation, and protein-protein interactions. It has primarily been studied in the nervous system. In muscle it has been implicated in **muscle regeneration**. | -4.3 | 2.32E-07 |
| GOLGA8R | golgin A8 family member R | A member of the **golgin** family of proteins, which are involved in **maintaining the structure and function of the Golgi apparatus** | -4.3 | 0.0005 |
| SCTR | secretin receptor | A **G-protein-coupled receptor** that primarily binds **secretin,** a peptide hormone involved in regulating various physiological processes. Its role in skeletal muscle is unclear, but it may be involved in energy metabolism. | -4.2 | 0.04 |
| LRRC38 | leucine rich repeat containing 38 | A protein that is part of the **leucine-rich repeat** family. It is involved in protein-protein interactions and **cell signaling,** which are essential for coordinating cellular processes. In muscle cells, these signaling pathways help regulate functions like **muscle contraction, fibre development**, and **maintenance.** | -4.1 | 2.85E-21 |
| MSS51 | MSS51 mitochondrial translational activator | Involved in responding to **mitochondrial stress. Its role in muscle is unknown.** | -4.1 | 5.11E-14 |

Table represents the log fold change values from baseline to 24h following an unaccustomed session of aerobic exercise (AE), together with a brief description of known function. Abbreviations: fc, fold change
