# Supplementary material for: Transcriptomic response of skeletal muscle to acute aerobic versus combined exercise in chronic kidney disease
Source: PLoS One. 2026 Feb 25;21(2):e0324303. doi: 10.1371/journal.pone.0324303 (PMC12935244; doi:10.1371/journal.pone.0324303)
Supplement: S2 Table — Table represents the log fold change values from baseline to 24h following an unaccustomed session of combined resistance and aerobic exercise (CE) together with a brief description of known function. Abbreviations: fc, fold change. (DOCX) [file pone.0324303.s002.docx]

Table S2. Top 20 Downregulated genes following CE

| Gene Symbol | Gene name | Gene function | Log2fc | Adj P value |
| --- | --- | --- | --- | --- |
| UNC13C | Unc-13 homolog .C | Involved in **synaptic vesicle priming** and **neurotransmitter release** at synapses. In muscle **UNC13C** helps in the release of **acetylcholine. It** is critical for efficient **neuromuscular transmission** and, ultimately, muscle contraction. | -2.4 | 0.01 |
| ABRA | Actin binding Rho activating protein | Plays a key role in the regulation of **actin cytoskeleton dynamics. In muscle it is involved in regulation of muscle contraction.** | -2.3 | 0.009 |
| FLRT3 | fibronectin leucine rich transmembrane protein 3 | Involved in cellular signaling and cell adhesion processes and is involved in **muscle development, muscle regeneration**, and **synapse formation.** | -2.2 | 0.02 |
| C2orf92 | chromosome 2 open reading frame 92 | Precise biological functions are not yet fully elucidated | -2.2 | 0.02 |
| SEC14L5 | SEC14 like lipid binding 5 | Associated with lipid-binding and regulation of lipid metabolism. Evidence of its function in muscle is limited. | -2.1 | 0.009 |
| U2AF1 | U2 small nuclear RNA auxiliary factor 1 | Plays a critical role in **RNA splicing** | -2.1 | 0.01 |
| NR4A1 | nuclear receptor subfamily 4 group A member 1 | A nuclear receptor protein that acts as a **transcription factor. It is involved in muscle hypertrophy.** | -2.1 | 0.0005 |
| ABCG1 | ATP binding cassette subfamily G member 1 | Involved in the **efflux of cholesterol** and other lipid molecules, helping to regulate lipid homeostasis in cells. In muscle it plays a crucial role in lipid homeostasis. | -2.0 | 0.002 |
| IDI2 | isopentenyl-diphosphate delta isomerase 2 | An enzyme that plays a role in **cholesterol biosynthesis** and the **mevalonate pathway.** | -1.9 | 0.03 |
| SMCO1 | single-pass membrane protein with coiled-coil domains 1 | Limited evidence on its biological function | -1.9 | 0.02 |
| MCF2L2 | MCF.2 cell line derived transforming sequence-like 2 | Limited evidence on its biological function | -1.8 | 0.04 |
| MAP2K6 | mitogen-activated protein kinase kinase 6 | An **activator of MAP kinases. In muscle, it activates p38 MAPK**, a critical regulator of **inflammatory responses** and **stress adaptation** in muscle cells. | -1.8 | 0.02 |
| AMHR2 | anti-Mullerian hormone receptor type 2 | A receptor protein that is part of the **TGF-β** superfamily. It has been implicated in **muscle regeneration.** | -1.8 | 0.006 |
| ALPK2 | alpha kinase 2 | A **protein kinase** that plays a role in intracellular signaling pathways, particularly those related to **immune responses** and **cellular stress**. | -1.8 | 0.02 |
| FRAS1 | Fraser extracellular matrix complex subunit 1 | A protein involved in the development of the extracellular matrix and basement membranes. In muscle it is involved in the formation of the **extracellular matrix.** | -1.7 | 0.04 |
| GTP2 | glutamic--pyruvic transaminase 2 | Plays a role in the regulation of various cellular functions, including signal transduction and metabolic processes. In muscle it is involved in growth and development, and in the response to injury. | -1.7 | 0.008 |
| PAMR1 | peptidase domain containing associated with muscle regeneration 1 | Encodes a protein with a role in cellular processes related to the regulation of protease activity, immune response, and tissue development. Its role in muscle is not well studied. | -1.7 | 0.04 |
| EIF3C | eukaryotic translation initiation factor 3 subunit C | Plays a key role in the process of **translation initiation** in protein synthesis as a component of the **eIF3 complex**, involved in the assembly of the translation machinery. This process is crucial for hypertrophy. | -1.7 | 0.007 |
| LMOD1 | leiomodin 1 | Plays a critical role in the **assembly and regulation of actin filaments in skeletal muscle and therefore in force development** | -1.7 | 0.03 |
| CFAP61 | cilia and flagella associated protein 61 | A protein that plays a role in the structure and function of **cilia** and **flagella**, cellular organelles involved in motility and signaling. Its role in muscle is unclear. | -1.7 | 0.006 |

Table represents the log fold change values from baseline to 24h following an unaccustomed session of combined resistance and aerobic exercise (CE) together with a brief description of known function. Abbreviations: fc, fold change
