# Supplementary material for: Transcriptomic response of skeletal muscle to acute aerobic versus combined exercise in chronic kidney disease
Source: PLoS One. 2026 Feb 25;21(2):e0324303. doi: 10.1371/journal.pone.0324303 (PMC12935244; doi:10.1371/journal.pone.0324303)
Supplement: S3 Table — Table lists top 20 genes showing the greatest upregulation (ranked by log2 fold change) in skeletal muscle from patients with chronic kidney disease (CKD) 24h post an unaccustomed bout of aerobic exercise (AE). Gene expression changes are presented as log2 fold change (log2fc) with Benjamini-Hochberg adjusted P values. For comparison, corresponding aerobic exercise induced gene expression responses in healthy individuals were obtained from the MetaMEx database [3] for the closest available timepoint to our biopsy collection, and are shown as log2fc and adjusted P values. A dash (-) indicated that no comparable MetaMEx data were available for that gene. Where multiple studies are cited, the meta-analytic summary statistic is shown. (DOCX) [file pone.0324303.s003.docx]

**Table S3. Top20 genes upregulated in CKD skeletal muscle following acute aerobic exercise and comparison with healthy control genes**

| Gene Symbol | CKD AE log2fc | CKD AE Adj P | Timepoint post exercise | Healthy aerobic exercise log2fc | Healthy control Adj P | Timepoint post exercise | Reference |
| --- | --- | --- | --- | --- | --- | --- | --- |
| CHI3L1 | 10.7 | 4.28E-09 | 24h | 0.28 | 0.61 | 48h | (1) |
| SAA2 | 8.6 | 2.90E-06 | 24h | 1.04 | 0.13 | 48h | (1) |
| PTX3 | 7.2 | 2.35E-06 | 24h | 0.57 | 0.32 | 48h | (1) |
| MT1A | 7.1 | 1.82E-16 | 24h | -0.18 | 0.91 | 48h | (1) |
| MYL10 | 8.4 | 0.04 | 24h | -0.12 | 0.83 | 48h | (1) |
| IL1RL1 | 7.0 | 0.0004 | 24h | -0.01 | 0.97 | 48h | (1) |
| SFN | 6.8 | 0.0002 | 24h | 0.22 | 0.66 | 48h | (1) |
| CCL19 | 6.3 | 0.04 | 24h | 0.78 | 0.16 | 48h | (1) |
| MFSD2A | 6.2 | 0.0001 | 24h | 0.19 | 1.0 | 48h | (1, 2) |
| SAA1 | 6.2 | 7.28E-06 | 24h | 0.58 | 0.26 | 48h | (1) |
| GAL | 6.1 | 4.86E-05 | 24h | 0.75 | 0.16 | 48h | (1) |
| LIPG | 5.7 | 4.35E-07 | 24h | 0.54 | 1.0 | 48h | (1, 2) |
| NIPAL4 | 5.5 | 0.0004 | 24h | 0.04 | 0.93 | 48h | (1) |
| HTR1B | 5.4 | 0.01 | 24h | 0.38 | 0.55 | 48h | (1) |
| SPOCD1 | 5.4 | 0.0007 | 24h | 0.04 | 0.94 | 48h | (1) |
| C3orf52 | 5.3 | 0.0009 | 24h | 0.18 | 0.71 | 48h | (1) |
| FCGR1A | 5.3 | 0.0008 | 24h | 0.46 | 0.26 | 48h | (1) |
| SOCS3 | 5.1 | 5.07E-19 | 24h | 0.53 | 0.29 | 48h | (1) |
| CELC4G | 5.1 | 0.005 | 24h | - | - | - | - |
| SELE | 5.1 | 0.0004 | 24h | -0.03 | 0.94 | 48h | (1) |

Table lists top 20 genes showing the greatest upregulation (ranked by log2 fold change) in skeletal muscle from patients with chronic kidney disease (CKD) 24h post an unaccustomed bout of aerobic exercise (AE). Gene expression changes are presented as log2 fold change (log2fc) with Benjamini-Hochberg adjusted P values. For comparison, corresponding aerobic exercise induced gene expression responses in healthy individuals were obtained from the MetaMEx database (3) for the closest available timepoint to our biopsy collection, and are shown as log2fc and adjusted P values. A dash (-) indicated that no comparable MetaMEx data were available for that gene. Where multiple studies are cited, the meta-analytic summary statistic is shown.
