# Supplementary material for: Transcriptomic response of skeletal muscle to acute aerobic versus combined exercise in chronic kidney disease
Source: PLoS One. 2026 Feb 25;21(2):e0324303. doi: 10.1371/journal.pone.0324303 (PMC12935244; doi:10.1371/journal.pone.0324303)
Supplement: S4 Table — Table lists top 20 genes showing the greatest upregulation (ranked by log2 fold change) in skeletal muscle from patients with chronic kidney disease (CKD) 24h post an unaccustomed bout of combined aerobic and resistance exercise (CE). Gene expression changes are presented as log2 fold change (log2fc) with Benjamini-Hochberg adjusted P values. As the CE exercise comprised both aerobic and resistance exercise components and combined exercise responses are not reported in the MetaMEx database, corresponding gene expression responses to aerobic exercise (AE) and resistance exercise (RE) in healthy individuals were obtained from MetaMEx for comparison. Healthy control data are shown as log2fc and adjusted P values. Where multiple studies are cited, the meta-analytic summary statistic is shown. (DOCX) [file pone.0324303.s004.docx]

**Table S4. Top20 genes upregulated in CKD skeletal muscle following acute combined exercise and comparison with healthy control genes**

| Gene Symbol | CKD CE log2fc | CKD CE Adj P | CKD AE Log2fc | CKD AE Adj P | Healthy aerobic exercise log2fc | Healthy control AE Adj P | Timepoint post AE | Reference | Healthy resistance exercise (RE) log2fc | Healthy control RE Adj P | Timepoint post RE | Reference |
| --- | --- | --- | --- | --- | --- | --- | --- | --- | --- | --- | --- | --- |
| SFN | 6.8 | 0.001 | 6.8 | 0.0002 | 0.22 | 0.66 | 48h | (1) | 0.13 | 1.0 | 24h | (4, 5) |
| MT1A | 5.8 | 0.01 | 7.1 | 1.82E-16 | -0.18 | 0.91 | 48h | (1) | -0.97 | 1.0 | 24h | (4) |
| CHI3L1 | 5.6 | 0.002 | 10.7 | 4.28E-09 | 0.28 | 0.61 | 48h | (1) | 0.01 | 1.0 | 24h | (4, 5) |
| ADAMTS17 | 4.8 | 0.01 | 4.8 | 6.67E-24 | -0.16 | 0.69 | 48h | (1) | 0.1 | 1.0 | 24h | (4, 5) |
| SOCS3 | 4.6 | 0.008 | 5.1 | 5.07E-19 | 0.53 | 0.29 | 48h | (1) | 0.02 | 1.0 | 24h | (4, 5) |
| LRP8 | 4.5 | 0.003 | 3.3 | 7.64E-05 | 0.48 | 1.0 | 48h | (1, 2) | 0.09 | 1.0 | 24h | (4, 5) |
| C3orf52 | 4.3 | 0.01 | 5.3 | 0.0009 | 0.18 | 0.71 | 48h | (1) | 0.49 | 1.0 | 24h | (4, 5) |
| PTX3 | 4.1 | 0.01 | 7.2 | 2.35E-06 | 0.57 | 0.32 | 48h | (1) | 0.05 | 1.0 | 24h | (4, 5) |
| UBASH3B | 4.0 | 0.0002 | 4.5 | 4.83E-11 | 0.85 | 1.0 | 48h | (1) | 1.08 | 1.0 | 24h | (4, 5) |
| GADD45A | 3.9 | 0.01 | 4.8 | 1.65E-21 | 0.46 | 0.48 | 48h | (1) | 0.59 | 1.0 | 24h | (4-6) |
| HMOX1 | 3.9 | 1.867E-13 | 4.1 | 7.38E-16 | 0.52 | 0.70 | 48h | (1) | 0.68 | 1.0 | 24h | (4, 5) |
| TNC | 3.8 | 7.08E-07 | 4.9 | 2.75E-05 | 0.16 | 0.85 | 48h | (1) | 1.44 | 1.0 | 24h | (4-6) |
| NNMT | 3.8 | 0.04 | 4.6 | 9.03E-23 | 0.25 | 0.85 | 48h | (1) | 1.26 | 1.0 | 24h | (4-6) |
| RUNX1 | 3.7 | 0.005 | 5.0 | 1.86E-20 | -0.17 | 1.0 | 48h | (1, 2) | 0.67 | 1.0 | 24h | (4, 5) |
| SERPINB1 | 3.7 | 0.005 | -1.03 | 1.0 | -0.21 | 0.96 | 48h | (1) | 0.8 | 1.0 | 24h | (4, 5) |
| ACTC1 | 3.6 | 4.90E-07 | 2.8 | 0.008 | -0.45 | 0.80 | 48h | (1) | 3.38 | 1.0 | 24h | (4, 5) |
| ARMC9 | 3.6 | 0.04 | 4.6 | 4.34E-05 | 0.30 | 1.0 | 48h | (1, 2) | 0.02 | 1.0 | 24h | (4, 5) |
| TCAF2 | 3.6 | 0.007 | 2.9 | 1.0 | 0.19 | 0.39 | 48h | (1) | 0.02 | 1.0 | 24h | (4, 5) |
| TIMP1 | 3.5 | 3.77E-07 | 4.0 | 1.0 | 0.71 | 0.44 | 48h | (1) | 1.68 | 1.0 | 24h | (4, 5) |
| SERPINE1 | 3.5 | 0.001 | 4.1 | 1.0 | 0.02 | 1.0 | 48h | (1, 2) | 0.11 | 1.0 | 5.1 | (4-6) |

Table lists top 20 genes showing the greatest upregulation (ranked by log2 fold change) in skeletal muscle from patients with chronic kidney disease (CKD) 24h post an unaccustomed bout of combined aerobic and resistance exercise (CE). Gene expression changes are presented as log2 fold change (log2fc) with Benjamini-Hochberg adjusted P values. As the CE exercise comprised both aerobic and resistance exercise components and combined exercise responses are not reported in the MetaMEx database, corresponding gene expression responses to aerobic exercise (AE) and resistance exercise (RE) in healthy individuals were obtained from MetaMEx for comparison. Healthy control data are shown as log2fc and adjusted P values. Where multiple studies are cited, the meta-analytic summary statistic is shown.

Neubauer O, Sabapathy S, Lazarus R, Jowett JB, Desbrow B, Peake JM, et al. Transcriptome analysis of neutrophils after endurance exercise reveals novel signaling mechanisms in the immune response to physiological stress. J Appl Physiol (1985). 2013;114(12):1677-88.

2. Mahoney DJ, Parise G, Melov S, Safdar A, Tarnopolsky MA. Analysis of global mRNA expression in human skeletal muscle during recovery from endurance exercise. Faseb j. 2005;19(11):1498-500.

3. Pillon NJ, Gabriel BM, Dollet L, Smith JAB, Sardón Puig L, Botella J, et al. Transcriptomic profiling of skeletal muscle adaptations to exercise and inactivity. Nature Communications. 2020;11(1):470.

4. Damas F, Ugrinowitsch C, Libardi CA, Jannig PR, Hector AJ, McGlory C, et al. Resistance training in young men induces muscle transcriptome-wide changes associated with muscle structure and metabolism refining the response to exercise-induced stress. Eur J Appl Physiol. 2018;118(12):2607-16.

5. Liu D, Sartor MA, Nader GA, Gutmann L, Treutelaar MK, Pistilli EE, et al. Skeletal muscle gene expression in response to resistance exercise: sex specific regulation. BMC Genomics. 2010;11:659.

6. Kostek MC, Chen Y-W, Cuthbertson DJ, Shi R, Fedele MJ, Esser KA, et al. Gene expression responses over 24 h to lengthening and shortening contractions in human muscle: major changes in CSRP3, MUSTN1, SIX1, and FBXO32. Physiological Genomics. 2007;31(1):42-52.
